# Quantification of the Cumulative Shading Capacity in a Maize–Soybean Intercropping System Using an Unmanned Aerial Vehicle

**DOI:** 10.34133/plantphenomics.0095

**Published:** 2023-11-10

**Authors:** Min Li, Pengcheng Hu, Di He, Bangyou Zheng, Yan Guo, Yushan Wu, Tao Duan

**Affiliations:** ^1^ College of Land Science and Technology, China Agricultural University, Beijing, China.; ^2^ School of Agriculture and Food Sustainability, The University of Queensland, St Lucia, QLD, Australia.; ^3^ Agriculture and Food, CSIRO, GPO Box 1700, Canberra ACT 2601, ACT, Australia.; ^4^ Agriculture and Food, CSIRO, Queensland Biosciences Precinct, St Lucia, QLD, Australia.; ^5^ College of Agronomy, Sichuan Agricultural University, Chengdu, China.; ^6^ Institute of Microelectronics of Chinese Academy of Sciences, Beijing, China.

## Abstract

In intercropping systems, higher crops block direct radiation, resulting in inevitable shading on the lower crops. Cumulative shading capacity (*CSC*), defined as the amount of direct radiation shaded by higher crops during a growth period, affects the light interception and radiation use efficiency of crops. Previous studies investigated the light interception and distribution of intercropping. However, how to directly quantify the *CSC* and its inter-row heterogeneity is still unclear. Considering the canopy height differences (*H_ms_*, obtained using an unmanned aerial vehicle) and solar position, we developed a shading capacity model (SCM) to quantify the shading on soybean in maize–soybean intercropping systems. Our results indicated that the southernmost row of soybean had the highest shading proportion, with variations observed among treatments composed of strip configurations and plant densities (ranging from 52.44% to 57.44%). The maximum overall *CSC* in our treatments reached 123.77 MJ m^-2^. There was a quantitative relationship between *CSC* and the soybean canopy height increment (*y* = 3.61 × 10^−2^×ln(*x*)+6.80 × 10^−1^, *P* < 0.001). Assuming that the growth status of maize and soybean was consistent under different planting directions and latitudes, we evaluated the effects of factors (i.e., canopy height difference, latitude, and planting direction) on shading to provide insights for optimizing intercropping planting patterns. The simulation showed that increasing canopy height differences and latitude led to increased shading, and the planting direction with the least shading was about 90° to 120° at the experimental site. The newly proposed SCM offers a quantitative approach for better understanding shading in intercropping systems.

## Graphical abstract

**Figure:**
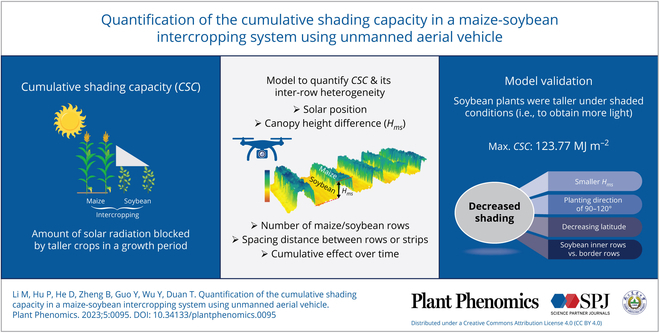

## Introduction

Intercropping refers to the planting pattern of 2 or more crops on the same land [1], which can increase yield production because of its advantages in suppressing weeds [2], reducing pests and diseases [3], and improving the use efficiency of light, water, and nutrients [4,5]. Taller crops intercept more photosynthetically active radiation (PAR) [6], but they also prevent direct solar radiation from reaching shorter crops [7]. This results in inevitable shading on shorter crops and affects their growth and development [8,9]. This study defined the amount of direct radiation blocked by taller crops as shading capacity (*SC*), and the cumulative shade capacity (*CSC*) as the accumulated *SC* over a period.

Shading has a substantial impact on competition among crops and microclimate in intercropping systems [10]. When shorter crops are subject to shading, their canopy is exposed to the variation of light quality (i.e., the ratio of red to far-red decreases) and the reduction of light quantity [11]. This change leads to alterations in the physiological [12] and phenotypic characteristics [13] of shorter crops. Shading increases the contents of chlorophyll *a* and *b* and decreases the Chl *a*/*b* ratio, as well as alters chlorophyll fluorescence, antioxidant activities, gas exchange, and radiation use efficiency (RUE) [14–16]. These alterations change the phenotypic characteristics of crops, including a reduction in stem diameter, leaf thickness, leaf area index, photosynthetic capacity, aboveground biomass, and yield [17–19], and an increase in plant height [16,20].

Although experiments in the field and controlled environments have qualitatively described and summarized the relationships between shading and phenotypic or physiological parameters (e.g., plant height) [21,22], direct quantification of the *CSC* in intercropping systems was rarely investigated. The existing methods can be classified into geometrical models and statistical models. Geometrical models, such as light competition, and interception models based on simplified geometric models and Beer’s Law were successfully applied to intercropping patterns of different species [23–25]. These models used an indirect method to quantify shading capacity by calculating the difference between the light interception of a maize–soybean intercropping system and that of monoculture system of soybean [26]. However, this method neglected the inter-row details that could play a critical role in intercropping, as light distribution might be more important than the total amount of light intercepted by intercropping systems [27]. A geometrical model was modified to investigate the border row effects on radiation interception, but they only provided estimations of instantaneous light capture by different intercropped rows [28,29]. Additionally, while the shading distance of maize was calculated based on plant height and sunlight, the relationship between shading distance and the amount of radiation was rarely investigated [30]. On the other hand, statistical models were constructed by regressing measured shading capacity and crop heights in a maize–soybean intercropping system [31]. However, these empirical models were strongly affected by the measured data and aimed to simulate instantaneous shading capacity by canopy height. Thus, it is necessary to develop a general model that can directly quantify the *CSC* and inter-row heterogeneity in intercropping systems.

Crop canopy height is an important input parameter for the aforementioned methods, which affects the interaction between intercropping crops, especially the light interception and competition of canopies [32,33]. In addition, the planting direction and latitude and longitude are also important factors affecting the intercropping light environment [34,35]. Of these factors, canopy height changes during the vegetative growing season, which requires continuous measurement. The traditional methods of obtaining canopy height (e.g., manual measurement by a ruler or telescopic leveling rod) are time-consuming, laborious, and/or destructive [36]. Recently, unmanned aerial vehicle (UAV) platforms had been used for high-throughput phenotyping of plant height under field conditions [36–38]. Moreover, UAV platforms were utilized to quantify the inter-row heterogeneity of crop growth status [39,40], which provided a technical reference for quantifying the effects of inter-row shading variation on shorter crop growth in intercropping systems.

The maize (*Zea mays* L.) and soybean (*Glycine max* (L) Merrill) strip intercropping system is a widely adopted practice in China due to its potential to obtain greater PAR interception and achieve higher yield production per unit area [41–43]. In shading research, soybean, as a rather shade-intolerant species, usually received more attention [44]. The novelty of our study lies in directly quantifying the *CSC* and its inter-row heterogeneity, and investigating the quantitative relationships between *CSC* and canopy height of soybean in maize–soybean intercropping systems. The objectives of the study were to (a) develop a quantitative shading capacity model (SCM) that considered the geometric relationship between canopy structure (i.e., the difference in canopy height between adjacent maize and soybean canopies) and solar position; (b) quantify the inter-row heterogeneity of *CSC* and the quantitative relationship between *CSC* and canopy height increment; and (c) evaluate the influence of model input parameters on shading to provide insights for optimizing intercropping planting pattern using the proposed SCM.

### Nomenclature

#### Symbols

**Table:** 

*r*	digital number of the red band
*g*	digital number of the green band
*b*	digital number of the blue band
*α*	solar elevation angle (°)
*β*	solar azimuth angle (°)
*δ*	solar declination (°)
*φ*	latitude (°)
*ω*	solar hour angle (°)
*N*	the day of the year
*LST*	local solar time
*R_a_*	direct solar radiation (MJ m^−2^ day^−1^)
*R_d_*	diffuse solar radiation (MJ m^−2^ day^−1^)
*R_g_*	global solar radiation (MJ m^−2^ day^−1^)
*R*_0_	extraterrestrial horizontal radiation (MJ m^−2^ day^−1^)
*I_sc_*	solar constant (=1,367 W/m^2^)
*ω_s_*	sunrise hour angle (°)
*E_0_*	sun–earth relative distance (km)
*S*	sunshine duration (h)
*S_0_*	maximum possible sunshine duration (h)
*D_s_*	shading distance (m)
*D_ms_*	row distance between adjacent maize and soybean strips (m)
*D_mr_*	distance from soybean rows to the original point (m)
*D_r_*	row spacing (m)
*Dr_ns_*	the difference range of north and south canopy height difference (m)
*γ*	planting direction (°)
*H_ms_*	canopy height difference between soybean strip and adjacent maize strips (m)
H_g_	the height of reference ground
*I_s_*	solar radiation intensity (MJ m^−2^)
*α_max_*	max solar elevation angle (°)
*SP*	shading proportion
*SC*	shading capacity (MJ m^−2^)
*CSC*	cumulative shading capacity (MJ m^−2^)
*SP_strip_*	the overall shading proportion on soybean strip
*SC_strip_*	the overall shading capacity on soybean strip
*H_i_*	the increment of canopy height (m)

#### Abbreviations

**Table:** 

SCM	shading capacity model
DAS	days after sowing
CHM	canopy height model
GCP	ground control point
GSD	ground sampling distance
DSM	digital surface model
EXG	excess green vegetation index
*R^2^*	coefficient of determination
RMSE	root mean square error (m)
RSE	residual standard error
AIC	Akaike information criterion

## Materials and Methods

### Field experiment

**Fig. 1.**
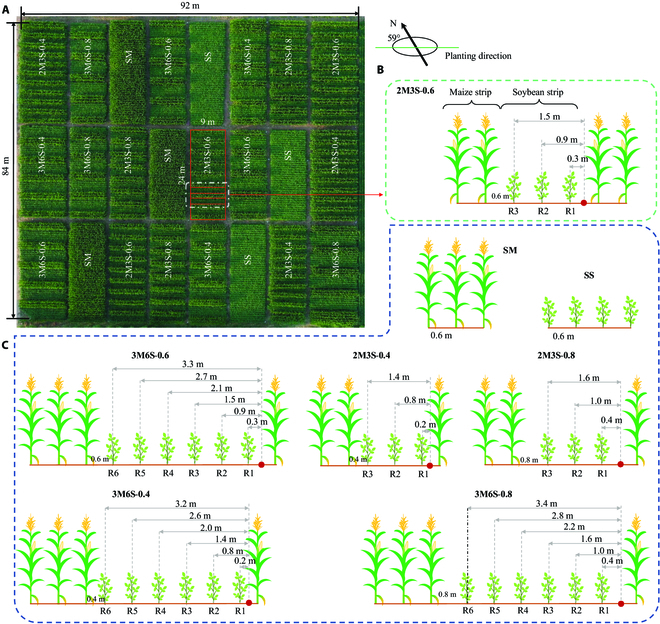
Field experiment layout of the maize–soybean intercropping system (A) and planting configurations of the strips (B) and the distance from a row to the adjacent south side maize strip (C). The field experiment included 8 treatments, each having 3 replicates. The intercropping treatments were the combinations of planting configurations (2M3S and 3M6S) and *D_ms_* (0.4 m, 0.6 m, and 0.8 m), and the sole maize (SM) and sole soybean (SS) for matched controls (A). The 2M3S-0.6 treatment was used as an example to demonstrate the strip of maize and soybean (B) and the *D_mr_* shading from the south side maize strip (C). See text for details.

A field experiment of a maize–soybean intercropping system was conducted at Lishu Experimental Station of China Agricultural University (43°16′N, 124°26′E), Jilin Province, China. The site is characterized as a subhumid climate, and the average annual temperature, precipitation, and sunshine duration are 6.9 °C, 626.7 mm, and 2,514 h (1992 to 2022), respectively. The soil is black soil with an average bulk density of 1.50 g cm^−3^. Maize (Zhengdan 958; hereafter, ZD958) and soybean cultivars (Jiyu 47; hereafter JY47) were sown on 2021 May 25 with the planting direction of 59° west of north (Fig. 1). The selected maize and soybean cultivars are widely sown in northeastern China.

The combination of narrow maize strips and wide soybean strips is widely adopted in China due to their suitability for machine operations using smaller-scale equipment [45]. Specifically, the planting configurations of 2 maize rows with 3 soybean rows (2M3S) and 3 maize rows with 6 soybean rows (3M6S) have been shown to achieve higher yields and land equivalent ratios [46,47]. Therefore, we selected the planting configurations of 2M3S and 3M6S for experimentation, with solo maize (SM) and solo soybean (SS) serving as control groups. The experiment was a randomized complete block design with 3 replicates (Fig. 1A).

For the 2 intercropping configurations (3M6S and 2M3S), there were 3 levels of the row distance between adjacent maize and soybean strips (*D_ms_*), i.e., 0.4, 0.6, and 0.8 m. These treatments hereafter were referred to as the combinations of intercropping configurations and *D_ms_* (i.e., 3M6S-0.4, 3M6S-0.6, etc.). Consequently, the experiment had a total of 8 treatments with 24 plots (3 replicates of each treatment). For all treatments, the row spacing (*D_r_*) within either the maize or soybean strip was 0.6 m, and the plant spacings for maize and soybean were 0.2 and 0.1 m, respectively. The field was 84 m wide and 92 m long, and each plot was 24 m wide and 9 m long. The adjacent plots were separated by a walkway (2 m wide). Figure S1 shows the monthly average temperature and total precipitation during the growing period of crops grown under rainfed conditions. Basal fertilizer was applied to both crops before sowing with 80 kg ha^−1^ N, 120 kg ha^−1^ P_2_O_5_, and 100 kg ha^−1^ K_2_O. Two top dressings of 80 kg ha^−1^ N each were applied to maize at the 8- and 14-completed leaf stage. The fertilization of the field experiment was uniform. Other field management practices (e.g., weed and pest control) were conducted according to local practices.

Maize and soybean strips were divided according to planting configuration and *D_ms_*. For instance, a maize strip was formed by 2 rows of maize (1.6 m wide), while a soybean strip consisted of 3 rows of soybeans (1.8 m wide) in the 2M3S-0.6 treatment (Fig. 1B). In the 3M6S configuration, soybean rows were labeled as R1 to R6 from south to north side. Similarly, for the 2M3S configuration, soybean rows were labeled as R1 to R3. The soybean canopy was shaded by maize on both the north and south sides due to changes in solar trajectory. The distance from soybean rows to the original point (details in “The shading distance at a specific time” section) of the south side maize strip (*D_mr_*, Fig. 1B and C) served as input parameters for the proposed SCM. It is worth noting that when the soybean canopy was shaded by the north side maize strip, the original point was the midpoint between the northern maize strip and the soybean strip, and *D_mr_* changed accordingly.

### Field measurements

Canopy heights of maize and soybean were measured at 4 growth stages of maize, i.e., at 41 (5-completed leaf), 48 (7-completed leaf), 56 (11-completed leaf), and 64 (tasseling) days after sowing (DAS). These dates corresponded to the growth stages of soybean of the fifth node, sixth node, beginning bloom, and full bloom, respectively (Table 1). For each strip in plots of intercropping treatments (i.e., 2M3S and 3M6S; Fig. 1B and C), plant heights (i.e., the vertical distance from the stem base to the highest position of the canopy) of 2 representative plants (i.e., it can reflect the average height of strip canopy) were manually measured with a telescopic leveling rod. For monoculture treatment (i.e., SM and SS; Fig. 1C), plant heights of 10 representative plants were measured for each plot. The average height of representative plants was counted as the canopy heights of the strip (intercropping treatments) or plot (monoculture treatments) [48]. The measurements of canopy heights were used to validate the UAV-derived canopy height model (CHM), which is described in detail below.

**Table 1.** Summary of UAV campaigns and field measurements for maize–soybean intercropping systems. The crops were sown on 2021 May 25. The relationship between thermal time and leaf number of maize is shown in Fig. S2.

Measurement date	Days after sowing	Maize stage	Soybean stage	Field measurement	UAV campaign
May 31	6	—	—	—	√
July 5	41	5-completed leaf	Fifth node	√	√
July 12	48	7-completed leaf	Sixth node	√	√
July 20	56	11-completed leaf	Beginning bloom	√	√
July 28	64	Tasseling	Full bloom	√	√

### High-throughput estimations of canopy height differences using UAV images

The canopy height difference between a soybean strip and its adjacent maize strips was another model input parameter for SCM. UAV campaigns were conducted to acquire images for establishing CHMs, which were then used for high-throughput estimations of canopy height differences. The workflow for estimating *H_ms_* followed 3 steps: (a) UAV data acquisition and preprocessing; (b) estimation of the strip canopy height of intercropping treatments using a CHM; and (c) calculation of the canopy height difference, which involved subtracting the canopy height of a soybean strip from that of adjacent maize strips.

**Fig. 2.**
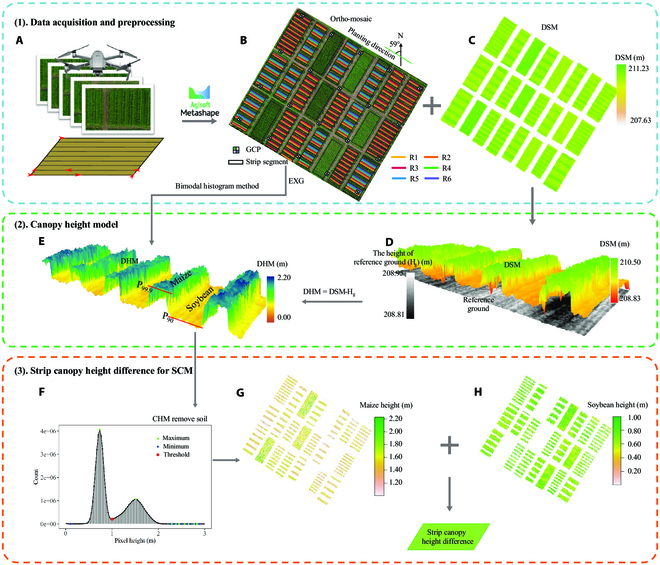
The workflow for high-throughput estimation of canopy height differences between soybean strip and the adjacent maize strips, including UAV image acquisition and processing (1), the establishment of canopy height model (CHM) (2), and the calculation of *H_ms_* (3). UAV image acquisition and processing were composed of UAV campaigns (A) and strip segmentations on UAV-derived digital ortho-mosaic (B) and digital surface model (DSM; C). The establishment of a CHM included the calculation of the difference between DSM and the reference ground (D) and the removal of soil and vegetative pixels of the other adjacent crop (E) from the CHM. Lastly, the strips of maize (G) and soybean (H) were separated by the threshold (F), and the canopy heights were then estimated from the corresponding CHMs, followed by the calculation of *H_ms_*.

UAV flights were conducted before emergence (i.e., 6 DAS) and on the same dates as canopy height measurements to capture time-series images of the whole field (Table 1). To mitigate the impact of weeds on subsequent processing, we performed weed removal before each UAV flight especially when the field is not fully covered by canopies. Before the first flight, a total of 20 ground control points (GCPs) were evenly distributed and fixed in the field. The geographic locations (longitude, latitude, and altitude) of these GCPs were measured by Stonex RTK-GNSS devices (Stonex Srl, Monza, Italy). A quadrotor UAV system, the Mavic 2 Pro (DJI Inc., Shenzhen, China) mounted with a 20-megapixel complementary metal oxide semiconductor (CMOS) sensor (5,472 × 3,648 pixels), was used to capture images at a flight altitude of 20 m, following the preplanned route (Fig. 2A). Both forward and side overlaps between images were 80%, and the cruising speed was set to 1.76 m/s. We deliberately avoided strong illumination and aimed to conduct UAV campaigns in diffuse lighting environments, regardless of whether it was sunny or overcast (excluding cloudy conditions), ensuring consistent lighting conditions during a flight mission. The image sequences were captured at a shooting interval of 2 s, with the total duration of each flight being about 12 min, resulting in about 530 images. The ground sampling distance of images was 0.48 cm/pixel.

The UAV image sequences were processed by a commercial photogrammetry software, Agisoft Metashape (Agisoft LLC, RU), with the GCPs for geometric correction and stitching images (Fig. 2B). The software generated digital surface models (DSMs), digital ortho-mosaic for each flight and reference ground (H_g_, defined as the DSM in the first flight on May 31). To extract the height of maize strips and soybean rows or strips from the DSMs, shapefiles of these strips and rows were created by segmenting them from the ortho-mosaic at the 11-leaf stage of maize as they were visually distinct at this stage. In addition, a soil mask was generated using the bimodal histogram method [49] to calculate the threshold values that were the local minimum of the excess green vegetation index (EXG; Eq. 1; [50]). The EXG, widely used in soil background removal [51,52], can effectively distinguish green vegetation in both sunny and overcast conditions [53].

EXG=2∗g−r−b (1)

where *r*, *g*, and *b* are the digital number of the red, green, and blue bands, respectively.

A UAV-derived CHM was built by the difference between DSM and the reference ground ([38]; Eq. 2; Fig. 2D) at each flight date (Table 1). The corresponding soil mask was applied to the CHM for removing soil and reserving vegetation pixels (Fig. 2E) for accurate estimations of canopy heights. After removing soil pixels, the pixels of adjacent maize (Fig. 2G) and soybean (Fig. 2H) strips, which might be overlapped by the maize strips, were further separated from the CHMs by using a thresholding method. The thresholds were the local minimum, which was determined from the histogram plotting the frequency distribution of height values of the CHMs (Fig. 2F), with 0.46, 0.78, 1.00, and 1.33 m for 41, 48, 56, and 64 DAS, respectively. The segmentation performance of maize and soybean on different growth stages are shown in Fig. S3. The canopy height was calculated as the 99.9th of the height values of the CHMs for a maize strip and the 90th of height values for a soybean strip (Fig. 2E) [54,55]. The CHM was used to estimate the canopy heights of maize and soybean strips (rows), which were interpolated using a cubic spline interpolation [56] to generate a smoother daily curve of their canopy heights. The daily average *H_ms_* was then calculated for 3 replications by subtracting the canopy height of a soybean strip from that of adjacent maize strips, and served as a model input parameter for the SCM (see below for details). The uncertainty caused by interpolation was ignored in this study.

$$\mathrm{CHM}=\mathrm{DSM}-H_{g}$$ (2)

### Calculation of solar position and direct radiation

Solar elevation angle (*α*; Eq. 3) and solar azimuth angle (*β*; Eq. 6) [57] were the inputs of the SCM:

$$\text{sin}\left(\alpha \right)=\text{sin}\left(\delta \right)*\text{sin}\left(\varphi \right)+\text{cos}\left(\delta \right)*\text{cos}\left(\varphi \right)*\text{cos}\left(\omega \right)$$ (3)

where *δ* is the solar declination (Eq. 4), *φ* is the latitude, and *ω* is the solar hour angle (Eq. 5).

$$\delta =23.45\times \text{sin}\left(360\times \frac{284+N}{365}\right)$$ (4)

$$\omega =15^{{}^{\circ}}\times \left(\mathrm{LST}-12\right)$$ (5)

β=arccossinδcosφ−cosδsinφcosωcosα,ifω<0∘360∘−arccossinδcosφ−cosδsinφcosωcosα,ifω>0∘ (6)

where *N* is the day of the year (i.e., the sequential day number starting with day 1 on January 1) and *LST* is the local solar time (24 h).

The shading on the soybean canopy in the maize–soybean intercropping system is primarily caused by the adjacent maize strip, which blocks direct solar radiation. In our study, the diffuse radiation was ignored as it is not sufficient to produce well-defined shading [58]. The direct solar radiation (*R_a_*) was calculated by subtracting diffuse solar radiation (*R_d_*) from global solar radiation (*R_g_*; Eq. 7):

$$R_{a}=R_{g}-R_{d}$$ (7)

Extraterrestrial horizontal radiation (*R*_0_; Eq. 8; [59]) was used to calculate *R_d_* (Eq. 8):

$$R_{0}=\frac{24\times 3,600}{\pi }I_{\mathrm{sc}}E_{0}\left(\text{cos}\left(\varphi \right)\text{cos}\left(\delta \right)\text{sin}\left(\omega_{S}\right)+\omega_{S}\text{sin}\left(\varphi \right)\text{sin}\left(\delta \right)\right)$$ (8)

where *I_sc_* is the solar constant (=1,367 W/m^2^), *ω_s_* is the sunrise hour angle (Eq. 9), and *E*_0_ is the sun–earth relative distance (Eq. 10):

$$\omega_{S}=\text{arccos}\left(-\text{tan}\left(\varphi \right)\text{tan}\left(\delta \right)\right)$$ (9)

$$E_{0}=1+0.033\text{cos}\left(\frac{2\pi N}{365}\right)$$ (10)

The diffuse fraction is the ratio of *R_d_* to *R_g_*, which has a strong correlation with the clearness index (*R_g_*/*R*_0_) and sunshine percentage (*S*/*S*_0_; [60,61]). The optimal general diffuse fraction model (Eq. 11) derived from [62] was used to calculate *R_d_* for the local experiment site (i.e., Jilin province).

$$\frac{R_{d}}{R_{g}}=1.06-0.56\times \frac{R}{R_{0}}-0.11\times {\left(\frac{R}{R_{0}}\right)}^{2}-0.26\times \left(\frac{S}{S_{0}}\right)-1.6\times {\left(\frac{S}{S_{0}}\right)}^{2}$$ (11)

where *S* is the sunshine duration from sunrise to sunset and *S*_0_ is the maximum possible sunshine duration (Eq. 12). *S* and *R_g_* were derived from the agrometeorological big data platform (http://www.wheata.cn/).

$$S_{0}=\frac{2\times \omega_{s}}{15^{{}^{\circ}}}$$ (12)

### Development of shading capacity model

**Fig. 3.**
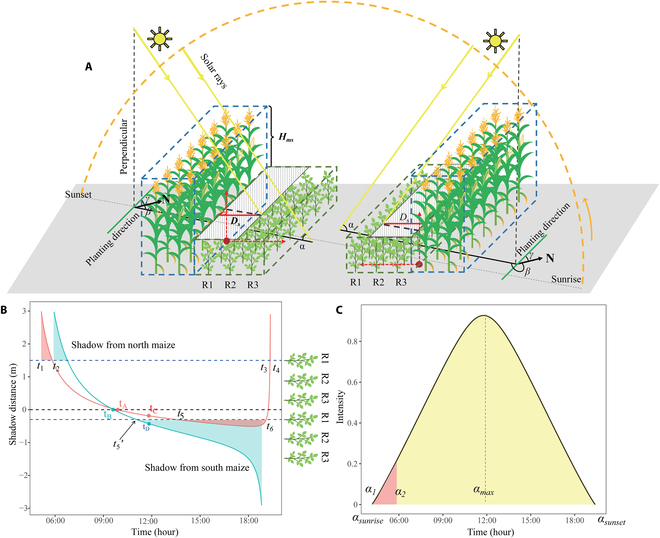
Schematic of the developed shading capacity model, including calculation of the *D_s_* at a specific time (A), calculation of daily cumulative shading time based on the dynamic curve of the *D_s_* (B), and simulation of shading proportion and shading capacity based on the relationship of the daily solar intensity over time (C). The 2M3S-0.6 treatment was used as an example to demonstrate the development process. See text for details.

The development of SCM was composed of 3 steps (Fig. 3), including (a) estimation of the shading distance (*D_s_*) according to the geometric relationship between *H_ms_* and solar rays (Fig. 3A), (b) estimation of the daily cumulative shading time on the soybean canopy (shaded area in Fig. 3B), and (c) determination of the shading proportion (*SP*) and shading capacity (*SC*; Fig. 3C). The initial input parameters are listed in Table 2.

**Table 2.** The inputs of the shading capacity model.

Name	Symbol
Solar elevation angle (°)	*α*
Solar azimuth angle (°)	*β*
Latitude (°)	*φ*
Direct solar radiation (MJ m^−2^ day^−1^)	*R_a_*
Planting direction (°)	*γ*
Canopy height difference (m)	*H_ms_*
Distance from soybean rows to the original point (m)	*D_mr_*

#### The shading distance at a specific time

In this study, maize and soybean strips were simplified into cuboid (i.e., blue and green dotted cuboids in Fig. 3A) and the radiation transmission through the canopy was ignored. A soybean strip was first shaded by the adjacent northern maize strip and then shaded by the other southern maize strip, depending on the solar position (Fig. 3A). A 2-dimensional coordinate system was established by taking the middle position between adjacent maize strip that forms shading and soybean strip as the original point. Taking 2M3S-0.6 as an example, when shading is caused by the northern maize strip, the *D_mr_* values for R1, R2, and R3 were 1.5 m, 0.9 m, and 0.3 m, respectively. Conversely, when shading came from the southern maize strip, the *D_mr_* values for R1, R2, and R3 were 0.3 m, 0.9 m, and 1.5 m, respectively. The vertical *Y*-axis was the direction of crop growth and the horizontal *X*-axis was within the plane of the ground surface and perpendicular to the planting direction. At a specific time, the *D_s_* of a maize strip on the adjacent soybean canopy was calculated using the *H_ms_*, solar position, and the planting direction (Fig. 3A; Eq. 13; [30,63]). The positive and negative signs of *D_s_* indicated the shading direction, and the absolute value of *D_s_* represents the dimension of *D_s_* on the soybean canopy.

$$D_{s}=\frac{H_{\mathrm{ms}}}{\text{tan}\left(\alpha \right)}\text{cos}\left(\beta +\gamma -90^{{}^{\circ}}\right)$$ (13)

where *γ* is the planting direction, starting from north and increasing counterclockwise; *γ* =0° meant that the planting direction was parallel to the north–south direction. In our experiments, the planting direction (*γ* = 59°) was obtained from the digital ortho-mosaic.

#### Daily cumulative shading time

The calculation of daily cumulative shading time was related to the *D_s_*. The *D_s_* from sunrise to sunset was calculated with a time interval of 1 min. During the vegetative growth stages of the intercropping systems in our study, there were 2 conditions (Fig. 3B and Fig. S4) to determine whether a row of the soybean strip was shaded by the adjacent maize strips at a specific time: (a) If *D_s_* was larger than 0, it meant that the shading was on the south side and the soybean strip was shaded by the adjacent northern maize strip; and if *D_s_* was less than 0, it indicated that the shading was on the north side and the soybean strip was shaded by the adjacent southern maize strip; *t_A_* and *t_B_* in Fig. 3B are the time that shading direction changed. (b) If |*D_s_*| was larger than *D_mr_* (Fig. 1B and C), the row was considered to be shaded. The daily cumulative shading time of a soybean row was the total time of the row shaded by the adjacent maize strips in a day. Take the 2M3S-0.6 treatment at 41 DAS as an example (Fig. 3B), the daily cumulative shading time of R1 comprised the total shading time from the northern maize strip (*t*_2_–*t*_1_ and *t*_4_–*t*_3_) and the southern maize strip (*t*_6_–*t*_5_).

#### Shading proportion and capacity on a soybean row

In addition to the daily cumulative shading time, the solar intensity in a day should be considered as well when evaluating the shading capacity on soybean canopy. Our study introduced the shading proportion (*SP*) to quantify the theoretical proportion of solar energy obscured by the adjacent maize canopy for a day. The solar radiation intensity (*I_s_*) is influenced by solar elevation angle, latitude, cloud cover, etc. For a given location, solar radiation intensity throughout the day is primarily affected by the solar elevation angle [64]. The relationship between *I_s_* and *α* is a sinusoidal function (Eq. 14; Fig. 3C; [65]). The value of *SP* was calculated by the ratio of the integral of sin (*α*) (Eq. 15), which was related to the chronological order of when the canopy began to be shaded (*t_s_*, *t*_5_, and *t*_5_′ in Fig. 3B) and the solar reached its maximum elevation angle (*t_m_*, *t_C_*, and *t_D_* in Fig. 3B):

$$I_{s}=\text{sin}\left(\alpha \right)$$ (14)

$$\mathrm{SP}=\left\{\begin{array}{c} \frac{\int_{\alpha_{1}}^{\alpha_{2}}\text{sin}\left(\alpha \right)\mathrm{d\alpha}}{\int_{\alpha_{\text{sunrise}}}^{\alpha_{\max}}\text{sin}\left(\alpha \right)\mathrm{d\alpha}+\int_{\alpha_{\text{sunset}}}^{\alpha_{\max}}\text{sin}\left(\alpha \right)\mathrm{d\alpha}},\;\text{if}\;t_{s}>t_{m}\\ \frac{\int_{\alpha_{1}}^{\alpha_{\max}}\text{sin}\left(\alpha \right)\mathrm{d\alpha}+\int_{\alpha_{2}}^{\alpha_{\max}}\text{sin}\left(\alpha \right)\mathrm{d\alpha}}{\int_{\alpha_{\text{sunrise}}}^{\alpha_{\max}}\text{sin}\left(\alpha \right)\mathrm{d\alpha}+\int_{\alpha_{\text{sunset}}}^{\alpha_{\max}}\text{sin}\left(\alpha \right)\mathrm{d\alpha}},\;\text{if}\;t_{s}<t_{m} \end{array}\right.$$ (15)

where *α*_1_ and *α*_2_ are the solar elevation angle for the row corresponding to the time of start shading (*t*_1_, Fig. 3B) and the time of end shading (*t*_2_, Fig. 3B), respectively. *α_max_* is the max solar elevation angle corresponding to *t_m_*. *α_sunrise_* and *α_sunset_* are the solar elevation angle at sunrise and sunset, respectively. The values of *α_sunrise_*, *α_sunset_*, and *α*_1_ are 0.

The shading capacity was then introduced to quantify the amount of the obscured solar direct radiation, which was calculated as the product of the direct radiation and shading proportion (*SP*) of the soybean row (Eq. 16). It should be noted that the calculation of *SC* was under the assumption that the weather conditions were consistent throughout the day. The overall *SP* and *SC* on a soybean strip were calculated from the *SP* (Eq. 17) and *SC* (Eq. 18) of each row, respectively. The (overall) *CSC* during a growth period was the sum of the (overall) *SC*.

$$\mathrm{SC}=R_{a}\times \mathrm{SP}$$ (16)

$${\mathrm{SP}}_{\text{strip}}=\frac{\sum_{i=1}^{n}{\mathrm{SP}}_{i}\times w_{i}}{\sum_{i=1}^{n}w_{i}}$$ (17)

$$SC_{\text{strip}}=\frac{\sum_{i=1}^{n}{\mathrm{SC}}_{i}\times w_{i}}{\sum_{i=1}^{n}w_{i}}$$ (18)

where *i* is the *i*th soybean row from the south side to the north side, and *w_i_*, *SP_i_*, and *SC_i_* are row width, *SP*, and *SC* of the row, respectively.

### Simulating the impact of inputs on overall shading proportion using SCM

Assuming that the field experiment and crop growth in our experiment were consistent across different latitudes and the planting direction was isolated from the effects of latitude and crop growth status [35], we investigated the effects of *H_ms_*, planting direction, and latitude on shading proportion variation using the SCM. Specifically, we varied the planting direction from 0° to 180° with an interval of 1°, and varied the latitude from 23°16′ N (approximately the Tropic of Cancer) to 53°16′ N (approximately the highest latitude of China) with an interval of 1°.

### Statistical analysis

A total of 4 UAV-derived CHMs were established at 4 growth stages (Table 1). The performance of the CHM was quantified by statistical criteria, including the coefficient of determination (*R^2^*; Eq. 19) and root mean square error (RMSE; Eq. 20). These criteria were computed with the canopy heights extracted by CHMs and the measured canopy heights. These validated CHMs were then used to estimate the canopy height difference and canopy heights of individual soybean rows of the corresponding stages.

$$R^{2}=\frac{\sum_{i=1}^{n}\;{\left({\hat{x}}_{i}-\bar{x}\right)}^{2}}{\sum_{i=1}^{n}\;{\left(x_{i}-\bar{x}\right)}^{2}}$$ (19)

$$\text{RMSE}=\sqrt{\frac{1}{n}\sum_{i=1}^{n}\;{\left({\hat{x}}_{i}-x_{i}\right)}^{2}}$$ (20)

where ${\hat{x}}_{i}$ is the canopy height estimated by the CHM, *x_i_* is the measured canopy height, *i* is the sequence of samples, and *n* is the number of samples.

The difference in *CSC* of different soybean rows among treatments was evaluated by analysis of variance. The least significant differences test was used to assess differences in *CSC* at the significance level of 0.05 (i.e., α = 0.05). A Spearman correlation was used to analyze the non-linear relationship between cumulative shading capacity and height increment of soybean rows at the α = 0.05 significance level, while logarithmic polynomial regression analysis was conducted to describe their relationship from the fifth-node stage to the full bloom stage (Table 1). The performance of the quantitative relational expression was evaluated with *P* values for the *F* statistic at a significance level of 0.05 (i.e., *α* = 0.05), residual standard error (RSE), and Akaike information criterion (AIC). The statistical and quantitative analyses were conducted in the R programming language (R Core Team, 2021) with customized scripts.

## Results

### Evaluation of canopy height estimation from UAV images

**Fig. 4.**
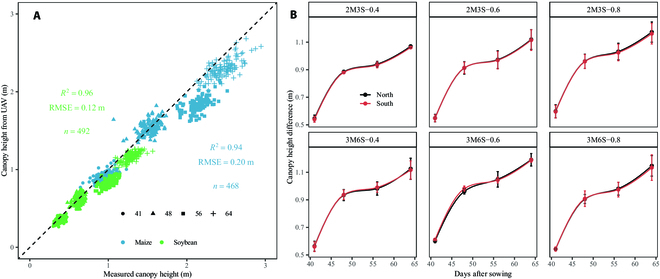
Comparison between measured canopy heights and UAV-derived canopy heights (A) and the changes of *H_ms_* over time (B) during the vegetative growth stages of soybean (green points) and maize (blue points) in the intercropping systems. Colors (B) represent the *H_ms_* on the north (black line) or south (red line) side, and the error bars represent the standard deviation of *H_ms_* across the 3 replicates for each of the 4 measurements.

The performance of canopy height estimation was evaluated by comparing it to corresponding measured canopy heights (Fig. 4A). Accurate estimations of canopy heights were obtained for both maize (*R*^2^ = 0.94 and RMSE = 0.20 m for 468 observations) and soybean (*R*^2^ = 0.96 and RMSE = 0.12 m for 492 observations) strips at different vegetative stages. The trend of UAV-estimated canopy height of maize and soybean strips is shown in Fig. S5. The *H_ms_* (Fig. 4B) had a similar pattern over time for different treatments. First, it increased rapidly until about 48 DAS (i.e., the elongation stage for maize and the fifth-node to sixth-node stage for soybean) and then plateaued, and the increase resumed from about 55 DAS (11-leaf to tasseling stage for maize and beginning bloom for soybean). The average increase rates (including the north and south sides) of stages 41 to 48 DAS, 48 to 56 DAS, and 56 to 64 DAS were about 0.05, 0.01, and 0.02 m/day, respectively. There was a little discrepancy of *H_ms_* on the south and north sides for each intercropping treatment. The difference range of the *H_ms_* in north and south (*Dr_ns_*) was from 4.59 × 10^−6^ to 2.15 × 10^−2^ m for all treatments. The variation of *H_ms_* on 4 measurements was slight among different replicates, with the standard deviation ranging between 4.50 × 10^−3^ and 9.28 × 10^−2^ m for all treatments.

### Quantitation of cumulative shading capacity and its inter-row heterogeneity on soybean

**Fig. 5.**
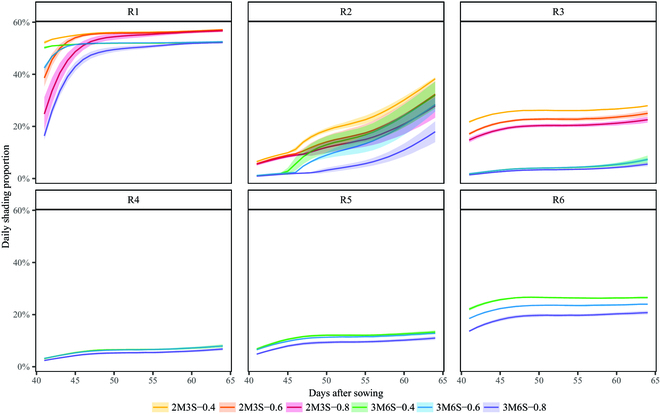
The dynamics of the daily shading proportion of solar energy obscured by the adjacent maize canopy on soybean rows in the intercropping treatments during the vegetative growth stages. The shaded area represents the standard deviation of 3 replicates of intercropping treatments.

The dynamics of shading distance (*D_s_*) of a maize strip on adjacent soybean rows varied across different growth stages (Fig. S4); it was affected by the canopy height difference of crops and the corresponding solar trajectory (Fig. S6). The dynamics of the daily shading proportion on the soybean canopy by an adjacent maize canopy was shown in Fig. 5. The variation trend of *SP* for R3 to R6 was insensitive to the day after sowing and the change of canopy height difference (Fig. 4B), while the *SP* of R2 was more sensitive to the canopy height difference and R1 rapidly increased to the upper limit (over 50%). The row of R1 observed the largest *SP* across all the intercropping treatments during the growth stages and the maximum value of *SP* reached 57.22%, 57.44%, 57.33%, 52.63%, 52.63%, and 52.44% for the 2M3S-0.4, 2M3S-0.6, 2M3S-0.8, 3M6S-0.4, 3M6S-0.6, and 3M6S-0.8 treatment, respectively (Fig. 5). The *SP* of R2 consistently increased during the growth stages. The other soybean rows observed minor variations in *SP* throughout the growth, with the average range of *SP* varied 8.74% and 6.24% for R3 in the 2M3S and 3M6S treatment, respectively. The average range of *SP* was 5.39%, 7.18%, and 6.42% for R4, R5, and R6 in the 3M6S treatment, respectively. Furthermore, soybean rows of 2M3S generally obtained a larger *SP* than those of 3M6S treatments. For soybean rows of the treatments with the same planting configurations, the *D_ms_* affected the *SP* on soybean rows. The same soybean rows of the treatments with smaller *D_ms_* typically obtained a larger *SP*, e.g., the R1 of 2M3S-0.4 observed a larger *SP* than 2M3S-0.6 and followed by 2M3S-0.8. In addition, similar daily cumulative shading time patterns on soybean rows were observed for the *SP* (Fig. S7).

**Fig. 6.**
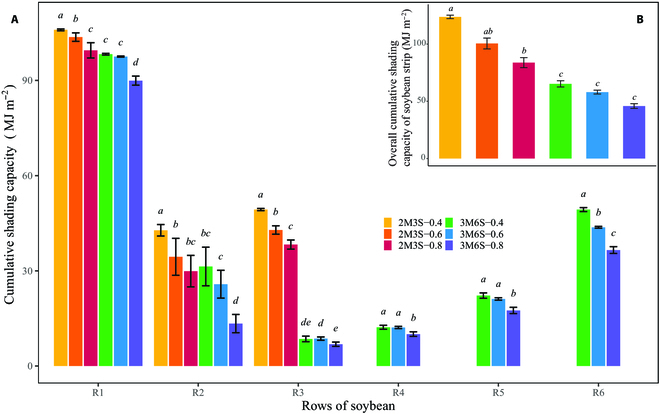
Comparison of the (overall) cumulative shading capacity for soybean rows (A) and strips (B) among intercropping treatments. Error bars represent the standard deviation of the 3 replicates of treatments. Italic letters (*a*, *b*, *c*, etc.) on the column bars indicate the significant difference (*P* < 0.05) of *CSC* among rows and strips of treatments.

The *CSC* varied among soybean rows of different intercropping treatments during our vegetative growth stages (Fig. 6A). The differences in the *CSC* among the 3 treatments corresponding with the narrower soybean strip (i.e., 2M3S) were more pronounced. The *CSC* of border rows in a strip was greater than those of inner rows for each treatment, i.e., the *CSC* of R1 and R3 was greater than that of R2 of the soybean strip in the 2M3S treatments and the *CSC* of R1 and R6 was larger than the other rows of soybean strip in the 3M6S treatments (e.g., for 2M3S-0.4, the *CSC* of R1, R2, and R3 was 105.95, 42.77, and 49.31 MJ m^−2^, respectively). The *CSC* of the same soybean rows differed significantly among planting configurations of intercropping. Soybean rows in the 2M3S treatments usually obtained greater values of *CSC* than the 3M6S treatments, i.e., the R1, R2, and R3 in 2M3S treatments had larger values of *CSC* than the corresponding rows in the 3M6S treatments (e.g., for R1, the *CSC* of 2M3S-0.4 and 3M6S-0.4 was 105.95 and 98.27 MJ m^−2^, respectively). For the same soybean rows of treatments with the same planting configurations, the rows of treatments with the smaller *D_ms_* usually observed larger values of *CSC* (e.g., for R1, the *CSC* of 2M3S-0.4, 2M3S-0.6, and 2M3S-0.8 was 105.95, 103.66, and 99.48 MJ m^−2^ respectively). Similarly, when aggregated to strip scale, the overall *CSC* of strips significantly differed among planting configurations (Fig. 6B). Soybean strips of 2M3S treatments had larger values of *CSC* than 3M6S treatments (e.g., the overall *CSC* of 2M3S-0.4 and 3M6S-0.4 was 123.77 and 65.25 MJ m^−2^, respectively). Soybean strips of treatments with smaller *D_ms_* suffered greater *CSC* (e.g., the overall *CSC* of 2M3S-0.4, 2M3S-0.6, and 2M3S-0.8 was 123.77, 100.52, and 83.83 MJ m^−2^, respectively).

**Fig. 7.**
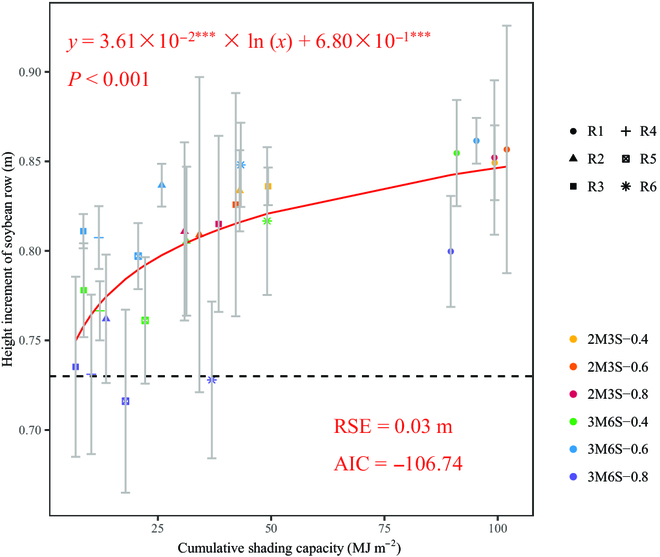
The relationship between cumulative shading capacity (*CSC*) and canopy height increment of soybean rows from the fifth-node stage (41 DAS) to the full bloom stage (64 DAS). The annotations (*p*, RSE, and AIC) serve as metrics for evaluating the performance of the quantitative relationship. The colors and shapes of the dots represent treatments and soybean rows, respectively. The error bars represent the standard deviation of 3 replicates. The dotted line represents the height increment of the sole soybean during the vegetative growth stage.

The canopy height increment of soybean rows from the fifth-node stage (41 DAS) to the full bloom stage (64 DAS) was positively correlated with the increasing of *CSC* during the same period, with a notable Spearman correlation coefficient of 0.76 (*P* < 0.001). The relationship was described by a logarithmic function (*y* = 3.61 × 10^−2^×ln(*x*)+6.80 × 10^−1^, *P* < 0.001, Fig. 7), with an RSE of 0.03 m and an AIC of −106.74. The R5 of 3M6S-0.8 and R1 of 3M6S-0.6 treatments observed the smallest (0.72 m) and largest (0.86 m) height increment on average. Compared to the canopy height increment (0.74 m) of the sole soybean treatment (without shading from maize), R5 and R6 of 3M6S-0.8 treatments obtained relatively smaller increments in canopy height.

### Evaluating the effects of inputs on overall shading proportion

**Fig. 8.**
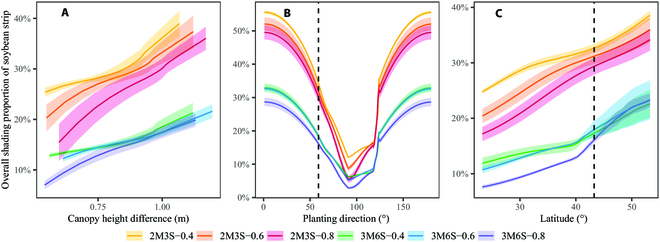
The changes of overall shading proportion on soybean strip (*SP_strip_*) over the changes of canopy height difference (A), planting direction (B), and latitude (C). The vertical *Y*-axis represents the daily average of *SP_strip_* during the growth period of our study in (B) and (C). The dotted lines represent the planting direction (B) and the latitude of the experiment in our field experiment (C), and the shaded area represents the standard deviation of 3 replicates.

The *H_ms_*, planting direction, and latitude notably affected the overall shading proportion of soybean strip (*SP_strip_*) estimated by the proposed model (Fig. 8). The *SP_strip_* consistently increased in response to the increasing *H_ms_* and latitude for all intercropping treatments (Fig. 8A and C). In general, the *SP_strip_* first decreased as the planting direction increased from 0 to 90° until the minimum *SP_strip_* was reached and then increased as the planting direction was further increased to 180° (Fig. 8B). Planting direction in the range from 90° to 120° (the exact direction of the local sunrise and sunset) can minimize the shading. With the changes of *H_ms_*, planting direction, and latitude, the 2M3S treatments observed a larger *SP_strip_* when compared to the 3M6S treatments. *D_ms_* had a more obvious impact on *SP_strip_* of the 2M3S treatments than those of the 3M6S treatments. For the treatments with the same planting configurations, the treatment with smaller *D_ms_* typically obtained a larger *SP_strip_* and vice versa.

## Discussion

Shading capacity or radiation capture in intercropping systems was commonly estimated by geometrical or empirical models based on field measurements of radiation and crop height. These models were subjected to inaccuracies of field measurements and were limited to describing the relationship between instantaneous PAR interception (or shading) and crop height [28,31]. This study developed a new model to directly quantify shading capacity and its inter-row heterogeneity of the soybean canopy in the maize–soybean intercropping system. The shading capacity model was applicable to different intercropping treatments with various latitudes and planting configurations (e.g., planting direction, strip width, and row spacing). Therefore, our model has the potential to serve as a powerful tool for optimizing the sowing date [66] and planting configuration [67] in intercropping systems across different regions.

The developed model can be used to simulate the inter-row heterogeneity of shading on soybean in the maize–soybean intercropping system (Figs. 5 and 6). The border-row soybean, especially in the southernmost row, tended to suffer more cumulative shading capacity than other inner rows in the canopy due to the border-row effect. This result was compatible with the lower PAR interception of the border row [4]. Shading from taller crops reduced the total radiation and increased the fraction of diffuse radiation on shaded crops in intercropping systems, which is the major reason for RUE increase of shaded crops [68,69]. Additionally, the inter-row heterogeneity of shading capacity also affected light interception and RUE of shaded crops [70], and crop growth exhibited sensitivity to changes in shading intensity and PAR [71,72]. Therefore, the quantitative simulation of cumulative shading capacity and its inter-row heterogeneity in this study will improve our understanding of the effects of shading on shaded crop growth and RUE in intercropping systems. Unlike instantaneous radiation interception or shading capacity, which was usually verified by the fraction of light interception (i.e., fPAR) [23,28], cumulative radiation interception was difficult to measure and verify directly. To validate the reliability of our simulation, we quantified the relationship between cumulative shading capacity and increment of soybean canopy height and found that soybean canopy heights increased under shaded conditions when compared with solo soybean (Fig. 7). The result was supported by previous studies [16,20,73] and also consistent with the observation that soybean border rows were taller than the inner rows (Fig. S8). These results illustrated the reliability of the proposed shading capacity model and the quantitative relationship in our study.

Row distance between adjacent maize and soybean strips and planting configurations were major factors that affected the shading capacity on soybean. A reduction in row distances and the use of narrower soybean strip widths increased shading capacity. In particular, the shading capacity of treatments with a narrow soybean strip width (i.e., 2M3S treatments) was found to be more sensitive to changes in row distance (Figs. 5 and 6). These findings were consistent with previous studies, which concluded that soybean strip width was a critical factor in determining radiation environment in maize–soybean intercropping systems [34].

The canopy height difference is the primary factor that contributes to shading in intercropping systems [74], with greater shading resulting from a larger difference in canopy height (Fig. 8A). A previous study has demonstrated that crop height has a notable impact on interspecific competition between cereals and soybeans, favoring weed control while impeding soybean growth [75]. Furthermore, differences in canopy height can alter the microclimate of intercropping systems, including factors such as wind speed, temperature, moisture, and light environment [76]. Making appropriate adjustments to these factors can be beneficial for enhancing intercropping performance [77]. The calculation of canopy height difference therefore directly affected the estimation of the cumulative shading capacity on soybean. UAV-derived canopy height difference did not always agree well with field-measured values at 4 growth stages (Fig. 4A); the discrepancy might be due to the subjectivity of manual measurements [78]. Moreover, soybean outperformed maize in terms of the estimation of canopy height by UAV; this might be attributed to the differences in their canopy characteristics. The soybean canopy was more “closed” compared to the more “open” canopy of maize [79], which also explained the difference in height statistical quantiles for maize and soybean canopies (Fig. 2E). We considered the canopy height difference between both sides (Fig. 4B) to explain the slight differences in growth status caused by microclimate changes [80] and enhance the applicability of the model.

The latitude and planting direction were other important factors for estimating shading capacity, as they influence the solar position and the relationship between solar position and canopy geometry structure, respectively [30,81–83]. The shading proportion decreased as the planting direction angle increased from 0° to 90° (Fig. 8B), which was supported by the study that PAR intercepted by soybean reduced in the same range of planting direction [34]. The shading proportion increased sharply at 120°, which corresponded to the direction of the local sunrise and sunset. This increase was attributed to the sharply increased shading distance on the border row (Fig. S4, shadow distance curve of 45 DAS). Shading was more prominent at higher latitudes, and relative irradiance decreased as latitude increased [35,82], which was reflected in the increasing shading proportion with latitude observed in this study (Fig. 8C). The findings suggested that planting direction should be considered in optimizing intercropping systems and that the effects of latitude on shading should be taken into account in regions with higher latitudes. These results have implications for the design and management of intercropping systems and can contribute to more efficient use of solar energy in crop production.

The proposed shading capacity model assumed that the weather changes were consistent throughout a day, and the relationship between solar radiation intensity and elevation angle followed a theoretical sinusoidal function (Fig. 3C). However, the cumulative shading capacity was estimated by multiplying shading proportion with direct solar radiation measurement, which worked on both clear and cloudy conditions and reduced the impact of this assumption to some extent due to the uncertainty of daily solar radiation [84]. Referring to previous studies on simulating the light environment in intercropping systems [68,85], we simplified the maize strip into a cuboid. However, due to the unavailability of detailed canopy structure information, the transmission of radiation within the maize canopy was ignored. The detailed 3-dimensional canopy architecture model of maize strips will be combined with the proposed shading capacity model to improve the accuracy of shading capacity quantification in our further study [86].

In the absence of water and fertilizer stress, light competition is the major limiting factor for crop growth and development in intercropping [87]. Consequently, we neglected the influence of water distribution and temperature when quantifying the effect of shading on soybean growth (Fig. 7). Shading plays a crucial role in the performance of intercropping systems [88], while overall and uniform radiation in intercropping canopy also markedly impacts the efficiency of the systems and crop growth [35]. Therefore, in future research, we will focus on the effect of overall PAR interception on crop growth of both maize and soybean in the intercropping system.

The shading capacity model can be used to further explore the quantitative effects of PAR or shading distribution on the phenotypic or photosynthetic parameters, such as leaf area index, aboveground biomass, chlorophyll content, and yield of shorter crops [19,20,89,90]. Coupling the shading capacity model with a crop model (e.g., Agricultural Production Systems sIMulator, APSIM) provides a feasible method to study the microclimate changes, crop competition, and resource utilization in intercropping systems [91,92]. Furthermore, high-resolution satellite imageries have gradually been used in crop classification and quantification of canopy structure of crops [93,94]. Therefore, the shading capacity model has the potential to be applied on a large spatial scale, in combination with multi-source remote sensing data.

This study proposed a shading capacity model that can quantitatively estimate the cumulative shading capacity and its inter-row heterogeneity of intercropping systems. For all intercropping treatments in our study, the cumulative shading capacity on the border row was always greater than that on the inner rows. The southernmost soybean row was most severely affected by shading, with shading proportion reaching over 50%. The model quantitatively described the logarithmic relationship between cumulative shading capacity and canopy height increment of soybean. Holding other factors constant, higher latitude regions had greater shading, and the planting direction of 90° to 120° resulted in the least shading in our field experiment. The model shows a high potential for optimizing intercropping planting patterns by adjusting planting configurations, planting direction, and variety selection. The shading capacity model only required the measured input parameters (i.e., canopy height difference), which can be efficiently acquired using the low-cost UAV system. Coupled with crop models or remote sensing data, the shading capacity model can be used to explore the effects of shading on crop growth, light competition, and microclimate in intercropping systems on a larger spatial scale in the future.

## Data Availability

The datasets analyzed in this study are freely available from the corresponding author upon reasonable request.

## Supplementary Materials

Supplementary 1 Fig. S1. The monthly average temperature and total precipitation during the growing stage of crops. Fig. S2. The relationship between thermal time and leaf number of maize. Fig. S3. The segmentation performance of maize and soybean on different growth stages. Fig. S4. Trend of shading distance (2M3S-0.6 as an example). Fig. S5. The change trend of UAV estimated canopy height of maize and soybean strips in different stages. Fig. S6. Schematic of shading direction during the period of this study, take July 15 as an example. Fig. S7. The trend of daily cumulative shading time in different rows among treatments; the shaded area represents the standard error of 3 replicates. Fig. S8. Soybean height inversed from UAV on 64 DAS in different rows.
