# Supplementary material for: Quantification of the Cumulative Shading Capacity in a Maize–Soybean Intercropping System Using an Unmanned Aerial Vehicle
Source: Plant Phenomics. 2023 Nov 10;5:0095. doi: 10.34133/plantphenomics.0095 (PMC10637764; doi:10.34133/plantphenomics.0095)
Supplement: Supplementary 1 — Fig. S1. The monthly average temperature and total precipitation during the growing stage of crops. Fig. S2. The relationship between thermal time and leaf number of maize. Fig. S3. The segmentation performance of maize and soybean on different growth stages. Fig. S4. Trend of shading distance (2M3S-0.6 as an example). Fig. S5. The change trend of UAV estimated canopy height of maize and soybean strips in different stages. Fig. S6. Schematic of shading direction during the period of this study, take July 15 as an example. Fig. S7. The trend of daily cumulative shading time in different rows among treatments; the shaded area represents the standard error of 3 replicates. Fig. S8. Soybean height inversed from UAV on 64 DAS in different rows. [file plantphenomics.0095.f1.zip › SM Figure captions.docx]

#

$$\begin{aligned} EXG=2*g-r-b\# LISTNUM myEq \end{aligned}$$

$$\begin{aligned} CHM=DSM-H_{g}\# LISTNUM myEq \end{aligned}$$

$$\begin{aligned} \sin\left( \alpha\right)=\sin\left( \delta\right)*\sin\left( \varphi\right)+\cos\left( \delta\right)*\cos\left( \varphi\right)*\cos\left( \omega\right)\# LISTNUM myEq \end{aligned}$$

$$\begin{aligned} \delta=23.45\times\sin\left( 360\times\frac{284+N}{365} \right)\# LISTNUM myEq \end{aligned}$$

$$\begin{aligned} \omega=15^{\circ}\times\left( LST-12 \right)\# LISTNUM myEq \end{aligned}$$

$$\begin{aligned} \beta=\left\{ \begin{aligned} \arccos\left[ \frac{\sin\left( \delta\right)\cos\left( \varphi\right)-\cos\left( \delta\right)\sin\left( \varphi\right)\cos\left( \omega\right)}{\cos\left( \alpha\right)} \right], if \omega<0^{\circ} \\ {360}^{\circ}-\arccos\left[ \frac{\sin\left( \delta\right)\cos\left( \varphi\right)-\cos\left( \delta\right)\sin\left( \varphi\right)\cos\left( \omega\right)}{\cos\left( \alpha\right)} \right], if \omega>0^{\circ} \end{aligned} \right.\# LISTNUM myEq \end{aligned}$$

$$\begin{aligned} R_{a}=R_{g}-R_{d}\# LISTNUM myEq \end{aligned}$$

$$\begin{aligned} R_{0}=\frac{24\times3600}{\pi}I_{SC}E_{0}\left( \cos\left( \varphi\right)\cos\left( \delta\right)\sin\left( \omega_{S} \right)+\omega_{S}\sin\left( \varphi\right)\sin\left( \delta\right) \right)\# LISTNUM myEq \end{aligned}$$

$$\begin{aligned} \omega_{S}=\arccos\left( -\tan\left( \varphi\right)\tan\left( \delta\right) \right)\# LISTNUM myEq \end{aligned}$$

$$\begin{aligned} E_{0}=1+0.033\cos\left( \frac{2\pi N}{365} \right)\# LISTNUM myEq \end{aligned}$$

$$\begin{aligned} \frac{R_{d}}{R_{g}}=1.06-0.56\times\frac{R}{R_{0}}-0.11\times\left( \frac{R}{R_{0}} \right)^{2}-0.26\times\left( \frac{S}{S_{0}} \right)-1.6\times\left( \frac{S}{S_{0}} \right)^{2}\# LISTNUM myEq \end{aligned}$$

$$\begin{aligned} S_{0}=\frac{2\times\omega_{s}}{15^{\circ}}\# LISTNUM myEq \end{aligned}$$

$$\begin{aligned} D_{s} =\frac{H_{ms}}{\tan\left( \alpha\right)}\cos\left( \beta+\gamma-90^{\circ} \right)\# LISTNUM myEq \end{aligned}$$

$$\begin{aligned} I_{s}=sin\left( \alpha\right)\# LISTNUM myEq \end{aligned}$$

$$\begin{aligned} SP=\left\{ \begin{aligned} \frac{\int_{\alpha_{1}}^{\alpha_{2}} sin(\alpha)d\alpha}{\int_{\alpha_{sunrise}}^{\alpha_{max}} sin(\alpha)d\alpha+\int_{\alpha_{sunset}}^{\alpha_{max}} sin(\alpha)d\alpha}, if t_{s}>t_{m} \\ \frac{\int_{\alpha_{1}}^{\alpha_{max}} sin(\alpha)d\alpha+\int_{\alpha_{2}}^{\alpha_{max}} sin(\alpha)d\alpha}{\int_{\alpha_{sunrise}}^{\alpha_{max}} sin(\alpha)d\alpha+\int_{\alpha_{sunset}}^{\alpha_{max}} sin(\alpha)d\alpha}, if t_{s}<t_{m} \end{aligned} \right.\# LISTNUM myEq \end{aligned}$$

$$\begin{aligned} SC = R_{a}\times SP\# LISTNUM myEq \end{aligned}$$

$$\begin{aligned} {SP}_{strip}= \frac{\sum_{i=1}^{n} {SP}_{i}\times w_{i}}{\sum_{i=1}^{n} w_{i}}\# LISTNUM myEq \end{aligned}$$

$$\begin{aligned} SC_{strip}= \frac{\sum_{i=1}^{n} {SC}_{i}\times w_{i}}{\sum_{i=1}^{n} w_{i}}\# LISTNUM myEq \end{aligned}$$

$$\begin{aligned} R^{2}=\frac{\sum_{i=1}^{n} \left( \hat{x}_{i}-\bar{x} \right)^{2}}{\sum_{i=1}^{n} \left( x_{i}-\bar{x} \right)^{2}}\# LISTNUM myEq \end{aligned}$$

$$\begin{aligned} RMSE=\sqrt{\frac{1}{n}\sum_{i=1}^{n} \left( \hat{x}_{i}-x_{i} \right)^{2}}\# LISTNUM myEq \end{aligned}$$

Supplementary

Fig. S1. The monthly average temperature and total precipitation during the growing stage of crops

Fig. S2. The relationship between thermal time and leaf number of maize.

Fig. S3. The segmentation performance of maize and soybean on different growth stages.

Fig. S4. Trend of shading distance (2M3S-0.6 as an example).

Fig. S5. The change trend of UAV estimated canopy height of maize and soybean strips in different stages.

Fig. S6. Schematic of shading direction during the period of this study, take July 15 as an example.

Fig. S7. The trend of daily cumulative shading time in different rows among treatments, the shaded area represent the standard error of three replicates.

Fig. S8. Soybean height inversed from UAV on 64 DAS in different rows.
